# Association of Interleukin-6 Gene Promoter Polymorphism with Coronary Artery Disease in Pakistani Families

**DOI:** 10.1155/2013/538365

**Published:** 2013-12-02

**Authors:** Humayoon Shafiq Satti, Sabir Hussain, Qamar Javed

**Affiliations:** Department of Biochemistry, Quaid-i-Azam University, Islamabad 45320, Pakistan

## Abstract

Interleukin-6 (IL-6) is a well-known inflammatory cytokine and suggested to be involved in the development of coronary artery disease (CAD). IL-6 gene expression has been investigated with controversy in CAD patients. This study investigates the association of the IL-6 gene expression with CAD, the molecular basis for the regulation of interleukin-6 expression in a Pakistani population. Our data show that the serum IL-6 levels were increased in patients with CAD compared with healthy controls and that the IL-6 gene polymorphism at -174 was more prevalent in CAD cases. There was a statistically significant association between the IL-6 gene polymorphism and CAD, which may be associated with an increased risk for the disease. Moreover, circulating IL-6 and hs-CRP levels were significantly higher in patients with CC genotype (*P* < 0.0001 and *P* < 0.0001, resp.). In a binary logistic-regression model, an independent association was found between CAD and increased serum IL-6 and hs-CRP levels and -174G>C polymorphism. This is the first report on the IL-6 expression and the IL-6 gene polymorphism in patients with CAD from Pakistan, and hence it highlights a novel risk factor for the disease.

## 1. Introduction 

Coronary artery disease (CAD) with an estimated 17.5 million deaths worldwide in 2005 is a leading cause of mortality from noncommunicable diseases. Countries with low-to-middle income share more than 80% of the global disease burden [1]. South Asians are among the highest susceptibility population with an alarmingly high incidence rate at younger age [2]. Although there are no absolute figures available as yet on morbidity and mortality related to CAD in Pakistan, the anticipated prevalence is inordinately high and severe [3]. According to the National Health Survey, every third Pakistani over the age of 40 years is hypertensive and 20% population of ≥60 years of age is facing hypercholesterolemia [4]. In addition to traditional CAD risk factors, strict socioracial marital customs, mass increase in urbanization, financial stress and adoption of westernization life style are among strong community-attributable risk factors for CAD in Pakistani population. But all of these risk factors alone do not fully explain the complexity or justify the magnitude of the recent explosion of cardiovascular incidence in Pakistani patients. Study of genetic regulation of the inflammatory processes, combined with knowledge of population specific risk factors, might improve prediction of familial and occupational risk of CAD.

Inflammation has an established role in atherosclerosis development and other manifestations of CAD. Inflammatory pathways are mediated by proximal cytokines such as interleukin-6 (IL-6) which is produced in various tissues, particularly in atherosclerotic lesion [5, 6]. Alwi and coworkers indicated that increased circulating IL-6 levels was associated with pathophysiology of acute coronary syndrome [7]. Furthermore, studies identified the increased risk of IL-6 in vascular biology and it has been linked with adverse effect in future events of myocardial infarction [8]. The most studied link of IL-6 with progression of atherosclerotic events is through the mediation of hepatic cells to produce an acute phase proteins in the response to develop atherosclerotic lesion in vascular intima [9, 10]. Circulating CRP itself has been shown to possess proatherogenic properties which might influence the propagation of atherosclerosis. High concentrations of CRP were present in atheromatous plaques and have been implicated in the propagation of coronary artery disease [11]. A genetic polymorphism at the position -174G>C of IL-6 gene and its association with development of CAD is controversial among various populations. Different population-based studies have shown that allele and genotype frequencies at this polymorphic site differ dramatically among racial and ethnic groups [12, 13]. It is perhaps the racial, ethnic, and geographical difference in the expression of inflammatory genes that predisposes Pakistani people to an early onset and severe forms of cardiovascular events, more than what is explained by traditional risk factors. Therefore we investigated the association of IL-6-174G>C polymorphism, circulating IL-6, and hs-CRP levels with CAD risk in native Pakistani families. This family-based case-control study was novel in our settings and has highlighted the link between inflammation, immunity, and CAD, thereby underscoring the biological insights gained from a genetic understanding of cardiovascular epidemic in South Asian population.

## 2. Methodology 

This is familial case-control association study, carried out in the Department of Biochemistry, Quaid-i-Azam University, Islamabad, from January 2012 to February 2013. Thirty indigenous Pakistani families with documented history of CAD in at least two successive generations were recruited from different regions of the country for this study. These families were ascertained from diseased proband. A total of 88 members from these families were enrolled after thorough pedigree analysis. Approval was obtained from Institutional Review Board (IRB), Quaid-i-Azam University. After obtaining written consent according to Helsinki Declaration of 1975 (revised in 1997) from all subjects, a detailed questionnaire was carefully filled through personal interview, done by a trained health professional. Patients were 36 with mean age of (46.4 ± 18.7) and healthy controls were 52 with mean age of (35.2 ± 17.4). Females were 44.4% in patients group and 42.3% in controls. The patients were confirmed on the basis of angiographic criteria established by François14 and electrocardiographic features. Criteria for hypertension were considered as the mean limit of systolic blood pressure was >139 mmHg and mean limit of diastolic blood pressure was >89 mmHg, measured 15 minutes apart or taking antihypertensive drugs. The category of overweight was defined as having BMI of 27 kg/m^2^ (kilogram per meter square) or greater. Control subjects were from the same ethnic region and their clinical histories were reviewed by a cardiologist being unaware of the objectives of study. The healthy controls representing same geographical location were included on the basis of normal electrocardiogram, normal angiography, and no history and symptoms of cardiovascular diseases.

Biochemical tests for quantitative analysis of serum lipids were performed using commercially available kits of AMP Diagnostics (Austria). Serum hs-CRP concentrations were measured by using a commercial high sensitivity turbidimetric kit provided by Roche Diagnostics Corp (Indianapolis, USA), whereas, circulating IL-6 level was measured using enzyme immunoassay (EIA) kit of Immunotech (Marseille, France). Biochemical assays were carried out according to the manufacturer instructions followed by standard enzymatic protocols.

**Figure 1:**
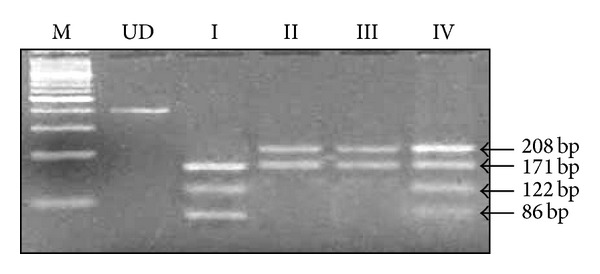
A representative 3% agarose gel photograph of IL-6-174G>C polymorphism stained with ethidium bromide: lane M represents DNA ladder (MBI Fermentas, UK). Lane UD represents undigested. Lane I corresponds to homozygote for the C allele (CC genotype) while lanes II-III correspond to homozygote for the G allele (GG genotype) and lane IV is heterozygote (GC genotype).

Genomic DNA extraction from blood samples was performed using standard organic method phenol-chloroform procedure. Amplification of 408 bp long promoter region was done by conventional PCR using specific primers 5′-GCG ATG GAG TCA GAG GAA AC-3′ (forward) and 5′-ATC TTT GTT GGA GGG TGA GG-3′ (reverse). The polymerase chain reaction was performed in the GeneAmp PCR System 9700 (Applied Biosystems Inc, FosterCity, CA). PCR protocol was adopted as described earlier [14]. After the purification of PCR products by using JET quick (purification spin/250 kit, GENOMED, Germany), 12 uL of PCR products from each sample was digested by 10 U of *NlaIII* restriction enzyme (MBI-Fermentas, England). At -174G>C position, C allele creates a cleavage site and cuts the fragment into four another fragments. Each fragment of heterozygous or homozygous genotype was separated into 208 bp, 171 bp, 122 bp, 86 bp, and 29 bp by using ethidium bromide stained 2% agarose gel, respectively (Figure 1).

Statistical analysis was performed by GraphPad Instat 3.05 (GraphPad Software Inc., San Diego, Calif). Basic and clinical variables like lipids, serum-IL-6, serum hs-CRP, age, and BMI of study population are mentioned as mean ± SD. Comparison of these variable between patients and control was carried out by chi-square and nonparametric Mann-Whitney test. Genotype of -174G>C and allele frequencies were measured by the chi-square and Fisher exact test, respectively. Analysis of variance was done among the -174G>C genotypes in patient group for IL-6 and hs-CRP using one way ANOVA. To evaluate possible relationship of -174G>C polymorphism, increased IL-6 and hs-CRP levels, binary logistic regression analysis was carried out by Minitab for Windows, version 14 (Minitab Inc. Chicago, USA). A significant probability (*P* < 0.05) was considered as a significant criterion.

## 3. Results 

Comparison of baseline characteristics and clinical variables between patients and control groups is shown in Table 1. In this study, patients were older in age than healthy members (*P* = 0.0050). Other demographic risk factors which were found significantly more prevalent among patients compared with healthy control include stress (*P* = 0.0001), physical activity (*P* = 0.0002), and hypertension (*P* < 0.0001). Whereas, BMI, smoking, and socioeconomic status were not significantly different among the members of both groups (*P* > 0.05, Table 1). Mean values of serum total cholesterol, triglycerides, cholesterol-LDL, and cholesterol-HDL levels were significantly associated with CAD patients (*P* < 0.05, Table 1). The mean circulating IL-6 levels were significantly increased in patients (3.9 ± 1.7) compared with control (3.0 ± 0.7) (*P* = 0.0268).

**Figure 2:**
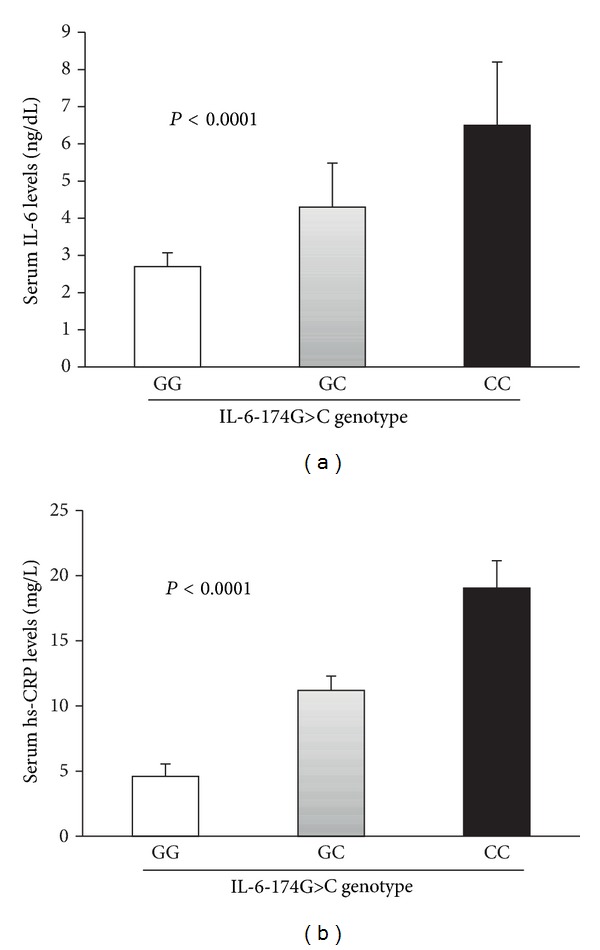
Influence of IL-6-174G>C polymorphism on the circulating levels of IL-6 and hsCRP in cardiovascular disease patients. Data represent mean ± SE. (a) Serum IL-6 levels, *P* < 0.0001, (b) serum hsCRP levels (*P* < 0.0001), in each genotype compared by 1-way-analysis of variance.

Genotype and allele distribution of IL-6-174G>C polymorphism is shown in Table 2. The frequencies of -174G>C genotypes were GG (20.4%), GC (12.4%), and CC (8.0%) in patients and GG (43.2%) and GC (16.0%) in controls. The CC genotype was absent in healthy subjects. The genotype of IL-6 at -174G>C in the patients and control population was in Hardy-Weinberg equilibrium (*P* = 0.0505 for CAD patients and *P* = 0.262 for healthy controls). The genotype distribution of IL-6-174G>C polymorphism in CAD patients showed a significant difference among the patients and control subjects (*P* = 0.0025; *P* value from 3 × 2 contingency table). The IL-6-174C variant allele was more prevalent in 34.7% patients compared with 13.5% in control subjects. There was a significant difference between the C and G allele frequencies in CAD cases and controls (OR = 3.4, 95% CI = 1.53–7.70, *P* = 0.0015, Table 2). The relationships of -174G>C polymorphism on serum IL-6 and hs-CRP levels are shown in Figure 2. Mean serum IL-6 and hs-CRP levels varied significantly among the IL-6 promoter genotypes within patient group. Carriers of CC genotype showed the highest levels of serum IL-6 and hs-CRP when compare with GC and GG genotypes (*P* < 0.0001, and *P* < 0.0001, resp., Figure 2). Binary logistic regression analysis for possible association of CAD with age, BMI, serum IL-6, serum hs-CRP, and -174G>C polymorphism is shown in Table 3. Four variables age (*P* = 0.008), serum IL-6 (*P* = 0.008), serum hs-CRP (*P* < 0.0001), and the -174G>C polymorphism (*P* = 0.029) were significantly associated with cardiovascular disease phenotype.

## 4. Discussion 

Although there is increasing evidence related to a central role of IL-6 in orchestrating the inflammatory cascades resulting in pathophysiology of CAD, the genetic regulation of its expression has been found to be complex. Several researchers have reported a potential influence of promoter polymorphisms on regulation of IL-6 at transcriptional level. But studies based on case-control association of -174G>C polymorphism with coronary events have shown conflicting results. To our knowledge there is no data available on genetic regulation of IL-6 in CAD patients in our population. Therefore, we questioned the association between CVD evidence and -174G>C polymorphism along with circulating IL-6 and hs-CRP levels in high-risk native Pakistani families.

In the current study, IL-6 promoter region polymorphism was significantly associated with CAD, and this association remained significant in multivariate analysis, even in the presence of confounding variables. Minor allele C at -174G>C was more prevalent (34.7%) in patients compared with healthy subjects (13.5%). Previous studies based on association of this polymorphism with different phenotypes of CAD have produced discrepant results. In some studies, this SNP has been associated with subclinical atherosclerosis events [15]. In agreement with our observations, the regional effect of IL-6-174G>C polymorphism was studied in patients with myocardial infarction from southern and northern Europe. Minor allele C was more prevalent (39.0%) in northern population compared with subjects from southern population [16]. However, some studies failed to prove any positive association between rs1800795 polymorphism of IL-6 gene and cardiovascular events in different ethnic groups [13, 17]. Ghazouani and colleagues identified the increased frequency of minor allele C in CAD patients (15.6%) compared with healthy control (14.3%). But they did not find any significant association of minor allele C with coronary artery disease in Tunisian patients [13].

In our patient group, a genotype-wise variation was observed in serum levels of hs-CRP and IL-6. The carrier of -174CC genotype patients represented the increased levels of circulating IL-6 and hs-CRP, respectively. Previous studies have recorded the influence of -174G>C polymorphism on circulating levels of hs-CRP and IL-6 [18, 19]. Vickers et al. declared baseline circulating hs-CRP as a significant heritable risk factor for cardiovascular system. Further, the CRP levels were significantly increased in carrier of -174CC of the IL-6 gene [19]. Similarly other studies demonstrated a significant association of IL-6-174G>C polymorphism along with increased levels of circulating serum IL-6 and hs-CRP with myocardial infarction and acute coronary syndrome (ACS) [16, 20, 21]. Further, this trend was supported by the observations from Turkey population. CC genotype showed the increased levels of both hs-CRP and IL-6 in thrombosis patients with positive history of infection [22]. This might reflect the complex physiological role of IL-6 and heterogeneity of disease subtypes and the populations studied. Therefore, our current family-based case-control study highlighted contribution of both accumulating CAD risk factors and genetic predisposition in incidence of CAD among high-risk Pakistani families.

## 5. Conclusion

In conclusion, we identified a significant association of IL-6-174G>C promoter region polymorphism with CVD in Pakistani patients. Further, the CC genotype contributes to increased levels of hsCRP and IL-6 in patients. This research has only scratched the surface and there is dire need for vigorous research in the high-risk populations for improved understanding of biological and sociobehavioral factors influencing the differential expression of this cytokine.

## Figures and Tables

**Table 1:** Baseline and clinical characteristics of study population *n* = 88.

Parameters	Patients (*n* = 36)	Control (*n* = 52)	*P* value
Age (years)	46.4 ± 18.7	35.2 ± 17.4	0.0050^a^
BMI (kg/m^2^)	25.9 ± 3.5	25.2 ± 3.5	0.2755^a^
Female/male (*n*)	16 (44.4%)/20 (55.6%)	22 (42.3%)/30 (57.7%)	0.8423^b^
Smokers/nonsmokers (*n*)	8 (22.2%)/28 (77.8%)	5 (9.6%)/47 (90.4%)	0.1825^b^
SES (low/high) (*n*)	23 (63.9%)/13 (36.1%)	31 (60%)/21 (40%)	0.8555^b^
Activity (low/high) (*n*)	21 (58.3%)/15 (41.7%)	9 (17.3%)/43 (82.7%)	0.0002^b^
Stressed/nonstressed (*n*)	27 (75%)/9 (25%)	16 (30.8%)/36 (69.2%)	0.0001^b^
Hypertensive/nonhypertensive (*n*)	22 (61.1%)/14 (38.9%)	7 (13.5%)/45 (86.5%)	<0.0001^b^
Systolic blood pressure (*n*)	147.5 ± 25.6	126.7 ± 14.6	<0.0001^a^
Diastolic blood pressure (*n*)	92.9 ± 12.5	82.8 ± 7.7	<0.0001^a^
Total-cholesterol (mg/dL)	198.2 ± 40.7	170.7 ± 28.4	0.0035^a^
Triglycerides (mg/dL)	134.4 ± 46.6	111.5 ± 26.4	0.0257^a^
LDL-cholesterol (mg/dL)	114.3 ± 30	97.5 ± 11.8	0.0295^a^
HDL-cholesterol (mg/dL)	38.0 ± 8.5	43.6 ± 8.1	0.0012^a^
Serum IL-6 (ng/dL)	3.9 ± 1.7	3.0 ± 0.7	0.0268^a^
Serum hsCRP (mg/L)	9.4 ± 5.7	3.0 ± 2.8	<0.0001

Values are given as means ± SD.

^a^ *P* values were calculated by using the Mann-Whitney test.

^b^ *P* values were calculated by the Pearson chi-square test.

**Table 2:** IL-6-174G>C genotype and allele frequencies of study population *n* = 88.

	Patients (*n* = 36)	Control (*n* = 52)	*P* value
IL-6-174G>C genotype			
GG	18 (20.4%)	38 (43.2%)	*χ* ^2^ = 11.9
GC	11 (12.4%)	14 (16%)	*P* = 0.0025^a^
CC	7 (8.0%)	—	

IL-6-174G>C allele frequencies			
G	47 (65.3%)	90 (86.5%)	*P* = 0.0015^b^ (C versus G allele)
C	25 (34.7%)	14 (13.5%)	OR = 3.4, 95% CI = 1.53–7.70

Values are given in percentage.

^a^ *P* value was calculated by chi-square test.

^b^ *P* value was calculated by the Fisher exact test. (OR: odds ratio for C and T allele frequencies in all subjects; CI: 95% confidence interval).

**Table 3:** Binary logistic regression analysis in all subjects when CVD was taken as a response variable.

Variables	*Z* value	Odd ratio	95% CI	*P* value
Age	2.66	0.95	0.91–0.99	0.008
BMI	0.50	0.96	0.81–1.14	0.615
Genotype: GG versus CG+GG	2.18	0.37	0.15–0.90	0.029
Serum IL-6	2.64	3.53	1.39–9.17	0.008
Serum hsCRP	4.16	0.49	0.35–0.69	<0.0001
